# The association between academic performance indicators and lifestyle behaviors among Kuwaiti college students

**DOI:** 10.1186/s41043-023-00370-w

**Published:** 2023-04-04

**Authors:** Ahmad R. Al-Haifi, Balqees A. Al-Awadhi, Nayef Y. Bumaryoum, Fahhad A. Alajmi, Rasha H. Ashkanani, Hazzaa M. Al-Hazzaa

**Affiliations:** 1 Department of Food and Nutrition Sciences, College of Health Sciences, PAAET, Showaikh, Kuwait; 2 Department of Home Economics, Basic Education Collage, PAAET, Showaikh, Kuwait; 3 Lifestyle and Health Research Center, Health Sciences Research Center, Princess Nourah Bint Abdulrahman University, Riyadh, Saudi Arabia

**Keywords:** Lifestyle behaviors, Academic markers, College students, Kuwait

## Abstract

**Background:**

Lifestyle behaviors are developed during young adulthood and carried through life. Accordingly, early detection of unhealthy behaviors can help prevent the increase in non-communicable diseases in the population. College students are an especially vulnerable group who, upon entering a new environment, tend to engage in unhealthy behaviors.

**Objective:**

The present study aims to evaluate the lifestyle behaviors of Kuwaiti college students and their association with markers of academic achievements.

**Methods:**

One thousand two hundred fifty-nine students participated in the present study and answered an online questionnaire pertaining to their sociodemographic status, academic performance indicators, body weight and height, and lifestyle behaviors. Data were collected from November 2020 to February 2021.

**Results:**

Results of the present study showed that obesity was significantly more prevalent among male participants than among females even though males were more physically active. Alternatively, females had a greater grade point average in college, slept more, and had more screen time. Interestingly, we were unable to detect a significant correlation between lifestyle behaviors and academic achievements.

**Conclusion:**

The observed differences in body mass index between genders may have been attributed to energy intake rather than energy expenditure.

**Supplementary Information:**

The online version contains supplementary material available at 10.1186/s41043-023-00370-w.

## Introduction

Lifestyle behaviors, such as eating habits, sleeping patterns, and physical activity, are daily practices that are molded by a combination of individual choices and social, cultural, and economic factors [1, 2]. World Health Organization (WHO) has recognized unhealthy lifestyles as a major cause of illness, mainly non-communicable diseases, and death [3, 4]. As such, monitoring lifestyles and implementing healthy behaviors in communities are of paramount importance. Interestingly, more often than not, behavioral patterns we established as young adults are carried through life, which makes college students a group of interest [5].

College marks a time of critical changes, where students have to adapt to a new environment and handle newfound responsibilities [6]. To cope with the transition, some students tend to engage in unhealthy behaviors such as physical inactivity [7–9], skipping breakfast [10, 11] poor diets [12, 13], sleep deprivation [14], etc. Several studies have documented the negative effects of unhealthy behavior, whereby 40% of premature deaths in the USA were reported to be attributed to unhealthy lifestyles [15].

Lifestyle behaviors are known to be influenced by several sociodemographic factors. Many studies investigated the relationship between gender and behavioral patterns [16–18]. Additionally, lifestyle behaviors were reported to vary between people of various economic statuses, in which those of lower income were shown to be engaged in more unhealthy lifestyle practices [19, 20].

In addition to unhealthy behaviors, the process of transition to college is usually accompanied by additional stress for students [21–23]. Stress stemming from college life can affect the academic performance of students [24, 25], which may subsequently influence their futures post-graduation [26]. Concurrently, academic performance in certain fields of study was shown to be affected by sociodemographic factors, such as gender [27, 28] and socioeconomic status [29–31]. Similarly, weight/diets and lifestyle behaviors seem to affect students’ academic standing [14, 32–35]. To our knowledge, limited information is available on the relationship between lifestyle behaviors and markers of academic performance among Kuwaiti students. Accordingly, it would be interesting to assess the impact of these factors on Kuwaiti college students. Thus, a study was designed in an attempt to assess the effect of some sociodemographic factors, *i.e.*, gender and geographic location, as well as weight on lifestyle behaviors and academic achievements of Kuwaiti college students.

## Materials and methods

A cross-sectional online study was performed, from November 2020 to February 2021, by approaching college students from various governorates across Kuwait. Ethical approval to perform the study was obtained from the Institutional Review Board (IRB) at the College of Health Sciences in Kuwait. Consenting students, who wished to participate in the study, were asked to fill out an online questionnaire.

The questionnaire was composed of several parts. One part focused on the sociodemographic status by including questions on gender, weight, height, marital status, governorate, and name of the college they were attending. As well as, their Grade Point Average (GPA) in college, which is the most common measure of academic performance, and their high school grade, which is usually expressed in percentages. Another part focused on the lifestyle behaviors of students and included questions about physical activity (22 questions), breakfast intake (1 question), sleeping habits (2 questions), and screen time (2 questions). The questionnaires were previously validated for adolescents and young people and were published elsewhere [36–39].

Students were also asked to enter their self-reported weight (Kg) and height (cm), and data were used for the calculation of Body-Mass index (BMI). The Centers for Disease Control and Prevention (CDC) BMI cutoff reference standards were used to classify underweight, normal weight, and overweight or obesity relative to the adult’s age [40].

### Statistical analysis

Data were analyzed using SPSS V.25 (Chicago, Illinois, United States). Descriptive statistics were performed on the anthropometric characteristics of the participants, and data were presented as mean ± standard deviation (SD) or as percentages (Table 1). Additionally, independent t test (for continuous dichotomous variables) and Chi-square test (for categorical variables) were used to assess significant differences among means relative to gender (Tables 1 and 2). Pearson correlation was used to correlate between lifestyle behaviors and high school grades and GPA. Due to the differences between genders for the measured parameters, a separate correlation was performed according to gender (Table 3). One-way ANOVA was performed to assess variations in the measured parameters for participants from different districts (Additional file 1: Table S1). Statistical significance was set at a *p* value < 0.05.

**Table 1:** Self-reported anthropometric characteristics of the participants

Variable	All	Male	Female	*p* value
Gender	N = 1259	577 (45.8%)	682 (54.2%)	**0.003**
Age (year)	21.5 ± 3.1	21.7 ± 3.1	21.4 ± 3.1	0.133
Marital status*				
Single	1130 (89.8%)	545 (94.6%)	585 (85.8%)	0.234
Body mass index (Kg/m^2^)	25.1 ± 6.0	25.9 ± 6.6	24.4 ± 5.4	** < 0.001**
BMI category**				
Underweight	109 (8.7%)	45 (7.8%)	64 (9.5%)	0.069
Normal weight	617 (49.4%)	257 (44.8%)	360 (53.4%)	** < 0.001**
Overweight	299 (24.0%)	138 (24.0%)	161 (23.9%)	0.183
Obesity	223 (17.9%)	134 (23.3%)	89 (13.2%)	**0.003**

*p* ≤ 0.05 are shown in bold

*N* number of participants

*N = 1258 participants

**N = 1248 participants

Data are presented as mean ± standard deviation of the mean or as frequency (percentage)

**Table 2:** Self-reported academic performance indicators and lifestyle behaviors among Kuwaiti college students

Variable	All	Male	Female	*p* value
High school grade (%)	75.1 ± 8.8	75.4 ± 8.5	74.9 ± 9.0	0.390
College GPA	3.0 ± 0.6	3.0 ± 0.6	3.1 ± 0.6	**0.041**
Breakfast intake (day/week)	4.0 ± 2.8	4.0 ± 2.7	3.9 ± 2.8	0.703
Total physical activity (METs-hours/week)	34.5 ± 42.2	40.8 ± 48.0	29.1 ± 35.8	** < 0.001**
Screen time (hours/day)	5.3 ± 3.7	4.7 ± 3.4	5.7 ± 3.9	** < 0.001**
Sleep duration (hours/night)	7.4 ± 1.6	7.3 ± 1.6	7.5 ± 1.6	**0.039**

*p* ≤ 0.05 are shown in bold

Data are presented as mean ± standard deviation of the mean

**Table 3:** Pearson correlation coefficients for selected variables with high school grade and college GPA

Variable		High school grade (%)		College GPA	
		*R*	*p* value	*R*	*p* value
Body mass index (Kg/m^2^)	Total	− 0.004	0.891	− 0.051	0.076
	Males	0.046	0.274	− 0.055	0.200
	Females	− 0.059	0.127	− 0.033	0.398
Breakfast intake (day/week)	Total	− 0.035	0.212	0.048	0.100
	Males	− 0.054	0.199	0.012	0.788
	Females	− 0.022	0.572	0.076	0.051
Total physical activity (METs-hours/week)	Total	0.054	0.057	− 0.031	0.290
	Males	0.041	0.333	− 0.066	0.120
	Females	0.064	0.098	0.026	0.499
Screen time (hours/day)	Total	0.031	0.281	0.034	0.240
	Males	0.035	0.400	− 0.005	0.898
	Females	0.033	0.385	0.049	0.214
Sleep duration (hours/night)	Total	− 0.045	0.116	0.002	0.947
	Males	0.004	0.921	− 0.058	0.177
	Females	− 0.081	**0.034**	0.044	0.255

*R* Pearson correlation coefficient

## Results

A total of 1259 students participated in the study and their basic reported anthropometric characteristics data are presented in Table 1. The number and percentage of female participants were significantly higher than those of males (54.2% females vs 45% males; *p* value = 0.003), but the age of participants was similar between genders at about 21.5 years. The majority of participants (~ 90%) were not married (single), and no significant differences were detected between genders. The mean BMI of the participants was about 25, in which that femals was significantly lower than that of males (*P* ˂0.001), this was mainly attributed to the high prevalence of obesity among males (23.3%) as compared to female (13.2%) participants (*p* = 0.003). It is worth mentioning that about 9% of participants suffered from being underweight.

Indicators of academic performance in relation to lifestyle behaviors are presented in Table 2. The average high school grade was similar between genders at about 75%, while college GPA was reported to be significantly higher (*p* = 0.041) for female (3.1 ± 0.6) as compared to male (2.9 ± 0.6) participants. The frequency (day/week) of breakfast intake was about 4 days/week, which was similar between genders. Total physical activity expressed as metabolic equivalents (METs) (hours/week) was reported to be significantly higher (p˂0.001) among male compared to female participants by about 10 METs (hours/week). In contrast, screen time of females was significantly higher (*p*˂0.001) than that of males by about 1 h per day, and sleep duration, expressed as hours per night, was reported to be modestly, though significantly (*p* = 0.039), higher by about 0.2 h per night in female participants.

The partial correlation coefficients between markers of academic performance and selected lifestyle factors are presented in Table 3. High school grades and college GPA did not significantly correlate with BMI. Likewise, the selected lifestyle factors, i.e., breakfast intake, total physical activity, sleep duration, and screen time, were not significantly correlated with the markers of academic performance (high school grade and college GPA).

Pearson’s correlation relative to gender reflected similar results. Although most measured variables showed a significant difference relative to gender, no significant correlation was detected between BMI, breakfast intake, physical activity, and screen time on one hand and high school grade and GPA on the other hand. Interestingly, the sleep duration of females was significantly and negatively correlated with their high school grades (*p* = 0.034) but not their college GPA.

Variations in academic achievements, BMI, and lifestyle behaviors across districts are presented in Table 1 (supplementary material). Results show that academic achievements varied significantly among districts. The high school grades of students in the Hawalli district (77.8 ± 10.1%) were significantly greater than the grades of students from the other districts (*p* value = 0.005). Similarly, college GPA of students in Hawalli district (3.1 ± 0.6) was significantly greater than the GPA of students in Al Jahraa (2.9 ± 0.6). Concurrently, there were no significant variations in BMI, breakfast intake, total physical activity, and screen time among districts (*p* value ˃ 0.05). Conversely, the sleep duration of students in Al Jahraa (7.6 ± 1.5 h/night) was significantly greater than that of students in Mubarak Al Kabir (7.1 ± 1.6 h/night).

## Discussion

Kuwait, a country in Western Asia, has benefitted from impressive economic growth in the past few decades with a 2.47% annual growth rate in Gross Domestic Product (GDP) from 1963 until 2020 [41]. Unfortunately, the economic development was paralleled with a drastic change in many lifestyle practices in a manner mimicking that of Western countries. This was highly pronounced by the adoption of a sedentary lifestyle, consumption of fast food, etc. Such changes in lifestyle behaviors are thought to affect all segments of the population including college students and it is not clear whether the changes in lifestyle behaviors had impacted academic performance.

In the present study, a high prevalence of overweight and obesity was reported. These results are in line with others [42–44] in which a high rate of overweight and obesity was reported among the Kuwaiti population. The observed high rate of obesity among male participants is in line with others [45], in which the rate of normal weight and overweight statuses was more prominent in Kuwaiti female college students whereas obesity was more abundant in males. Even though traditionally, plumpness was valued in Arab countries and considered a feminine trait [46], female college students are believed to have been influenced by the widely propagated images advertising thin models as the “perfect type” and are thus becoming more conscious of their weights. In line, women at a young age were reported to be very cautious about their body image and are likely to participate in weight loss or control activities [46, 47]. However, the observed low physical activity and increased screen time among women imply that their weight control measures are mainly related to dieting (reduction in energy intake) rather than increased physical activity (increase in energy expenditure). Future studies should focus on investigating the eating habits of Kuwaiti college students relative to gender.

Levels of overweight and obesity statuses exceeded those reported among university students in European countries [48]. Al-Isa [49] noted that while in Europe societal norms pressure people to remain thin, Kuwaiti people seem to accept obesity in the absence of societal penalties against fatness. El Ghazali, Ibrahim [50] attributed high BMIs among Kuwaiti college students to several factors (including consumption of sweets and adopting sedentary lifestyles) and noted that the regular consumption of fast food seems to be the most important factor. Not to mention that traditional Arab clothing helps hide the body shape and thus might play a role in altering the perceived body type and making students less interested in monitoring their weights.

In support, physical activity among university students in Saudi Arabia [51] and the USA [52] found that male students were more physically active than females. Although male students spent more time being physically active, they were also more obese while more females had a normal weight (Table 2). These results suggest that the difference in weight between the two genders is highly attributed to eating habits rather than physical activity. Interestingly, an often-overlooked problem is that 8.7% of the students are underweight, a classic case of the coexistence between obesity and undernutrition. This may relate to the pocket of extreme poverty or some form of disturbed eating behaviors and requires further investigation to address this serious issue.

Correlation analysis showed no differences in markers of academic performance among participants of varied BMI in both genders. The relation between BMI and academic performance is far from clear. Some studies reported an inverse relationship between BMI and academic performance [33, 53, 54], while others failed to detect any relationships [55, 56]. The relation may be confounded by several factors including weight-related psychological factors such as having low self-esteem; depression (caused by weight-biased treatment), etc. Factors are known to affect the academic performance of the students thus leading to lower academic performance.

Likewise, the relation between breakfast intake and academic performance is far from clear [57], though a positive relation was commonly reported. The inconsistency between studies may partially relate to the nutritional composition of the consumed breakfast. Thus, the failure to detect a relationship between breakfast intake and markers of academic performance may in part relate to the nutritional composition of the ingested breakfast. Therefore, future studies should focus on assessing the nutritional composition of breakfast among participants.

Moreover, physical activity is thought to improve cognitive function and brain structure and function [58]. However, its impact on academic performance is controversial and is confounded by several factors including the type, intensity, setting, etc. [59]. For instance, moderate physical activity performed during school hours seem to positively impact academic performance. The questionnaire from the present study was not detailed enough to capture the aforementioned factors and this may have been behind the failure to detect a significant correlation between physical activity and markers of academic performance. Future studies should give attention to details related to the varied aspects of physical activities to clarify any potential relationship.

Several studies have reported an inverse relationship between screen time and academic performance [60]. However, the present study failed to detect a significant correlation between these variables. It is important to note that this questionnaire did not differentiate between screen time spent on schoolwork and screen time spent relaxing and having fun. Whatever, a positive trend was observed although failed to reach statistical significance.

On the other hand, although the relationship between sleep duration and academic performance is extensively studied, the results remain to be non-conclusive. For instance, a review by Musshafen, Tyrone [61] reported that academic performance is correlated with sleep quality but not sleep duration. While, Dewald, Meijer [62] reported a moderate yet significant association between sleep duration, quality, sleepiness, and academic performance. Additionally, a positive association was reported between academic performance, duration, quality, and consistency of sleep patterns [63]. With the exception of females’ high school grades, where a significant negative correlation was observed, the present study failed to detect a significant correlation between sleep duration and academic performance. It is important to note that the only factor considered in the present study was the duration of sleep and that future studies should consider factoring in other variables such as the quality, and consistency of sleeping patterns. Additionally, future studies should look into more consistent methods of evaluating sleeping patterns rather than counting on self-reported data.

Pearson’s correlation was performed to detect a relationship between physical activity, sleep duration, and screen time (data not shown). Interestingly, we failed to detect a correlation between physical activity and sleep duration, which could indicate that the physical activities performed were not of high intensity. Alternatively, screen time was found to be positively correlated with sleep duration (*p* value ˂ 0.001) and negatively correlated with physical activity (*p* value ˂ 0.001). The present results suggest that the more time the students are spending on screen time, the more they sleep and the less they are physically active.

Overall, our study found no correlation between lifestyle behaviors and markers of academic achievements (Table 3). Previous studies have highlighted the importance of adopting healthy lifestyles on academic achievements [32, 64]. The observed minimal variation in the markers of academic achievement may have been behind the inability to detect a significant association with the varied component of lifestyle behaviors. Nonetheless, males seem to be more physically active and spend less time watching television (TV) or using social media and computers while females slept more and had a lower BMI.

In an attempt to investigate the effect of geographic location on academic achievements and lifestyle behavior of the participants, we studied these variables relative to the district the participants came from. Although some differences were observed among districts, mainly in academic achievement indicators and sleep duration (Additional file 1: Table S1), the data collected in the questionnaire do not provide enough information to explain the results. We speculate that the variations could be caused by differences in the socioeconomic status, mainly income and occupation, of the participants’ families. Studies have shown that improved socioeconomic status is associated with better academic performance [29–31]. Unfortunately, the questionnaire did not include questions about the economic situation of the students and thus no clear-cut conclusions can be made.

The present study showed that in general, Kuwaiti college students adopted healthy lifestyle behaviors, being physically active (34.5 h/ week), sleeping for more than 7 h daily, and spending on average 5.3 h/ day watching TV or using social media. It is worth noting, however, that students should improve their breakfast intake (4 days/ week). Concurrently, the lifestyle behaviors did not seem to affect students’ academic performance. The study also showed that females have a lower BMI than males, which might have positively affected their college GPA; however, more research regarding the eating habits of students should be performed. Regardless of the effect of lifestyle behaviors on academic performance, it is of paramount importance to promote healthy patterns among young adults to help prevent or decrease the occurrence of non-communicable illnesses. Lastly, it is important to note that the present study is not a cohort study and accordingly, longitudinal, observational studies may be needed to better assess the long-term effects of lifestyle behaviors on college students in Kuwait.

## Supplementary Information

**Additional file 1.** Supplementary files: Table S1.

## Data Availability

All data generated or analyzed during this study are included in this published article. Any additional data will be available from the corresponding author upon reasonable request.
