# Supplementary material for: The association between academic performance indicators and lifestyle behaviors among Kuwaiti college students
Source: J Health Popul Nutr. 2023 Apr 4;42:27. doi: 10.1186/s41043-023-00370-w (PMC10071657; doi:10.1186/s41043-023-00370-w)
Supplement: Supplementary file 1 — Additional file 1. Supplementary files: Table S1. [file 41043_2023_370_MOESM1_ESM.docx]

Supplementary material

Supplementary Table S1. Variation of selected academic achievements indicators and lifestyle behaviors among Kuwaiti college students relative to districts.

| Variable | All | Capital | Hawalli | Frwaniah | AlJahraa | AlAhmadi | Mubarak Kabir | *p*-value |
| --- | --- | --- | --- | --- | --- | --- | --- | --- |
| High school grade (%) | 75.1 ± 8.8 | 75.3 ± 8.7^b^ | 77.8 ± 10.1^a^ | 74.7 ± 8.7^b^ | 74.9 ± 8.6^b^ | 74.3 ± 7.7^b^ | 74.4 ± 9.4^b^ | **0.005** |
| College GPA | 3.0 ± 0.6 | 3.1 ± 0.6^a,b^ | 3.1 ± 0.6^a^ | 3.0 ± 0.6^a,b^ | 2.9 ± 0.6^b^ | 3.1 ± 0.6^a,b^ | 3.1 ± 0.6^a,b^ | **0.017** |
| Body mass index (Kg/m^2^) | 25.1 ± 6.0 | 25.9 ± 7.2 | 25.5 ± 5.6 | 24.7 ± 5.4 | 24.8 ± 5.5 | 25.5 ± 6.3 | 24.7 ± 6.0 | 0.089 |
| Breakfast intake (day/week) | 4.0 ± 2.8 | 3.7 ± 2.8 | 3.8 ± 2.8 | 4.0 ± 2.8 | 4.2 ± 2.7 | 4.1 ± 2.8 | 3.8 ± 2.9 | 0.315 |
| Total physical activity (METs-hours/week) | 34.5 ± 42.2 | 33.3 ± 42.7 | 33.7 ± 41.4 | 33.6 ± 43.2 | 37.3 ± 43.3 | 30.5 ± 36.8 | 38.3 ± 46.0 | 0.435 |
| Screen time (hours/day) | 5.3 ± 3.7 | 4.8 ± 3.3 | 5.2 ± 3.7 | 5.4 ± 3.7 | 5.7 ± 3.9 | 5.0 ± 3.7 | 5.3 ± 4.1 | 0.155 |
| Sleep duration (hours/night) | 7.4 ± 1.6 | 7.2 ± 1.7^a,b^ | 7.4 ± 1.6^a,b^ | 7.4 ± 1.7^a,b^ | 7.6 ± 1.5^a^ | 7.4 ± 1.7^a,b^ | 7.1 ± 1.6^b^ | **0.017** |

Data is presented as mean ± standard deviation of the mean.

Values in the same column with different superscripts (a,b) are significantly different from each other (p < 0.05).
